# Restricted intramolecular rotation of fluorescent molecular rotors at the periphery of aqueous microdroplets in oil

**DOI:** 10.1038/s41598-020-73980-7

**Published:** 2020-10-08

**Authors:** Jooyoun Kang, SangMoon Lhee, Jae Kyoo Lee, Richard N. Zare, Hong Gil Nam

**Affiliations:** 1 Center for Plant Aging Research, Institute for Basic Science, Daegu, 42988 Republic of Korea; 2 Department of Chemistry, Stanford University, Stanford, CA 94305 USA; 3 Department of New Biology, DGIST, Daegu, 42988 Republic of Korea

**Keywords:** Chemistry, Physical chemistry, Excited states

## Abstract

Fluorescent molecular rotor dyes, including Cy3, Cy5, and Alexa Fluor 555, dissolved in micron-sized aqueous droplets (microdroplets) in oil were excited, and the fluorescence intensity was recorded as function of time. We observed lengthening of the fluorescence lifetime of these dyes at the water–oil periphery, which extended several microns inward. This behavior shows that intramolecular rotation is restricted at and near the microdroplet interface. Lengthened lifetimes were observed in water microdroplets but not in microdroplets composed of organic solvents. This lifetime change was relatively insensitive to added glycerol up to 60%, suggesting that solution viscosity is not the dominant mechanism. These restricted intramolecular rotations at and near the microdroplet periphery are consistent with the reduced entropy observed in chemical reactions in microdroplets compared to the same reaction conditions in bulk solution and helps us further understand why microdroplet chemistry differs so markedly from bulk-phase chemistry.

## Introduction

Aqueous microdroplets (approximately 1–30 µm in diameter) exhibit unique physicochemical properties that drive the acceleration of various chemical reactions by the factor of 10^3^ or more compared to bulk solution^1–8^. Even more interestingly, the thermodynamic properties of chemical reactions in microdroplets are also altered to allow thermodynamically unfavorable reactions such as phosphorylation of sugars and redox reactions to occur spontaneously without an added energy source^9–13^. The interface of an aqueous microdroplet has different properties from a planar water interface^14–17^. It appears that a strong electric field exists at the microdroplet interface, although the magnitude remains to be firmly established^18–22^. In addition, vibrational spectroscopy suggested that the interface of a water nanodroplet shows significantly stronger hydrogen bonds than that of the planar liquid hydrophobic/water interface^14^. Moreover, the fluorescent dye rhodamine 6G in aqueous microdroplet has been shown to be localized and aligned near a water–oil interface, showing that translational and intramolecular rotations are restricted in the microdroplet environment^15^. These observations provide a possible explanation of why the kinetics and the thermodynamics of chemical reactions in microdroplets differ from the ones in bulk solution. These findings further motivated us to explore whether molecular motions such as internal molecular rotation are perturbated in microdroplets.

For this investigation, we monitored the behavior of intramolecular motion in microdroplets by measuring the fluorescence lifetime of electronically excited molecular rotors. Fluorescence lifetime imaging microscopy (FLIM) has been used for mapping the local mechanical properties of small, confined volumes such as microbubble shells and aerosols^16,17,23^. We employed several molecular internal rotor fluorescent molecules, including Cy3, Cy5, and Alexa Fluor 555 (AF555). These internal rotor molecules undergo rapid *cis*–*trans* isomerization, which results in loss of energy through nonradiative processes, leading to shortening the fluorescence lifetime^24^. Thus, the change in the microenvironment that restricts the intramolecular rotational movement, such as high viscosity, polarity, and temperature, can be estimated by examining the lengthening of fluorescence lifetime of an internal rotor molecule^25^.

## Results and discussion

### Fluorescence lifetime lengthening of cyanine dyes in aqueous microdroplets

First, we tested Cy3 dye, an open-chain trimethine cyanine dye, which contains a conjugation bridge capable of *cis*–*trans* isomerization (Supplementary Fig. S1a) as a probe for detecting changes in the intramolecular rotation in the bulk solution. We measured the lifetime of Cy3 dyes in the bulk aqueous solution containing different concentrations of glycerol, which induces lengthening of the lifetime of fluorescent molecular rotors, mainly, by increasing the viscosity of solution which slows down the rate of intramolecular rotation^25^. The lifetime of Cy3 gradually increased from 0.18 ns in bulk water to 1.8 ns in 100% glycerol, showing that Cy3 is an effective probe for intramolecular rotation. The relative change in the intramolecular rotation can be estimated from the acquired calibration curve (Supplementary Fig. S1b).

**Figure 1:**
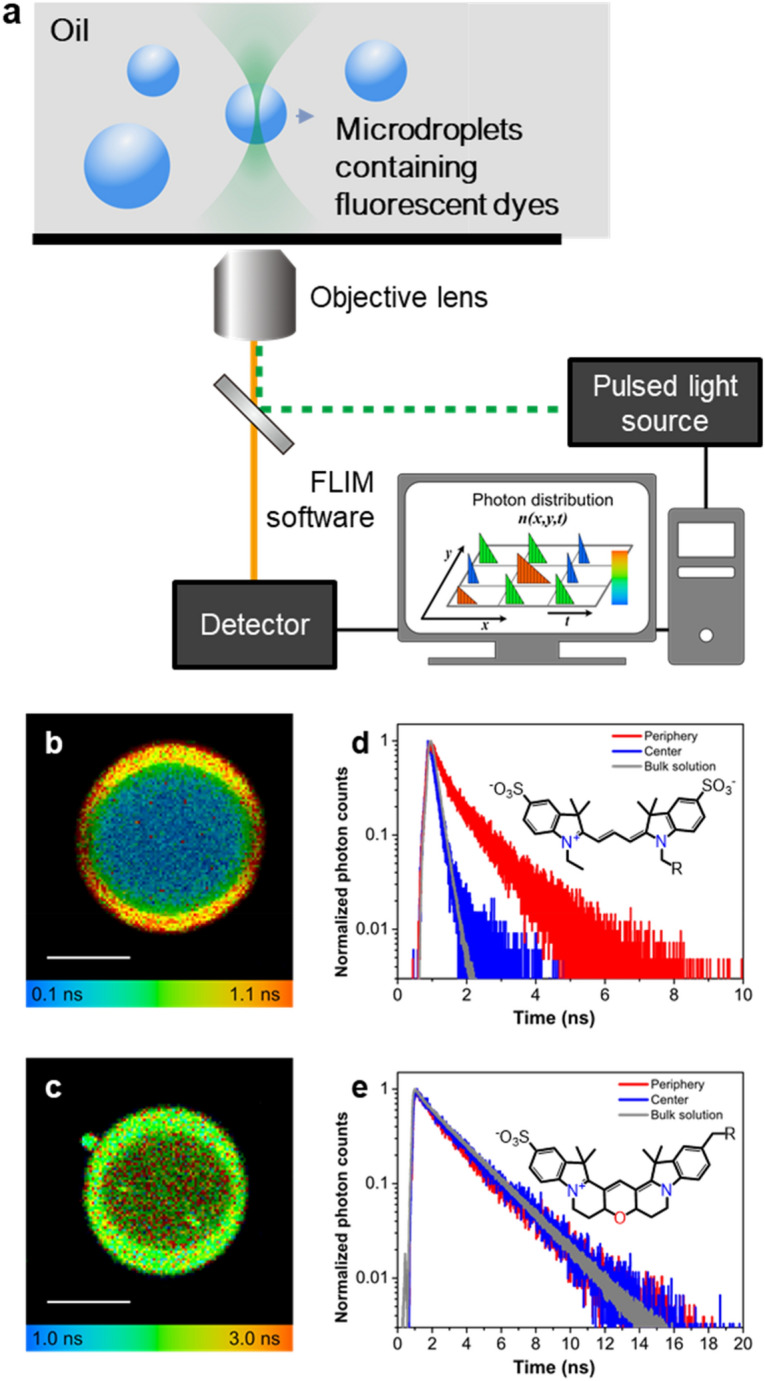
Cy3 fluorescent lifetime at the periphery of aqueous microdroplets. (**a**) Schematic of the experiment setup. (**b**, **c**) The fluorescence lifetime imaging microscopy (FLIM) images of Cy3 (**b**) and Cy3B (**c**). Scale bar, 5 µm. (**d**, **e**). Fluorescence decay curves of Cy3 and Cy3B at the central region (blue) and the peripheral region (red) of aqueous microdroplets. Plotted in gray are fluorescence decay curves of Cy3 and Cy3B in bulk water. The inset of each graph presents the chemical structure of the fluorophore.

To measure the change of the intramolecular rotational movement in microdroplets, we prepared microdroplets containing 1-µM Cy3 dye by ultrasonically emulsifying the mixture of water containing Cy3 dye and immersion oil (1:10 ratio, v/v). Figure 1a shows the schematic of the experimental setup for imaging of microdroplets. The solution containing aqueous microdroplets in oil was mounted on a glass coverslip and imaged with a confocal microscope equipped with FLIM measurement instrumentation. To avoid possible physical or chemical influence of the glass surface on both microdroplet and the bulk solution, we carried out the imaging of microdroplets located at least 4 μm above the coverslip.

Figure 1b shows a FLIM image and fluorescence lifetime data of Cy3 in an aqueous microdroplet that has a radius of approximately 5 μm. The fluorescence lifetime of Cy3 at the peripheral region of aqueous microdroplets exhibited a much-increased fluorescence lifetime (red, τ_periphery_ = 0.86 ns) compared to the central region (blue, τ_center_ = 0.17 ns) and in bulk water solution (grey, τ_bulk_ = 0.18 ns) (Fig. 1b,d). These data show that the intramolecular rotation of Cy3 is highly restricted at the periphery of oil-confined water microdroplets. We further compared the fluorescence decay temporal curves of Cy3 in bulk water and the central and peripheral regions of microdroplets. The decay curves were fitted by either a single- or bi-exponential function, depending on the shape of the decay curve. The decay curves in bulk water and the central region of the microdroplets were similar to each other, and they were fitted to a single exponential decay curve. However, the decay curves at the periphery of microdroplets were different from those in bulk water or in the central region of microdroplets. These decay curves were slower and appeared to have multi-exponential behavior. The data shown is the result of fitting to the bi-exponential function. The result showed that the fluorescence lifetime characteristic of Cy3 has changed in the peripheral region.

By comparing the increased fluorescence lifetime (τ_periphery_ = 0.86 ns) at the periphery of water microdroplets and the calibration curve shown in Supplementary Fig. S1b, the degree of the intramolecular rotation at the periphery of microdroplets is equivalent to that of a 65 wt% bulk glycerol solution. From the comparison of the viscosity value of the 65 wt% glycerol solution (~ 16 mPa∙s at 20 °C) with that of water (~ 1.0 mPa·s at 20 °C), it can be assessed how resistant the water near the periphery region of microdroplets is for intramolecular rotation. In contrast, the lifetime of Cy3 at the central region remained almost unaffected. This result shows that the degree of the restriction of Cy3 in microdroplets depends strongly on the radial location inside the microdroplet.

In order to confirm that the increase in the fluorescence lifetime was caused by the restriction of intramolecular rotation, we employed Cy3B dye, which has the same common chromophores as Cy3 but has a rigid backbone; therefore, Cy3B has a fixed conformation (Supplementary Fig. S1a)^26^. Unlike Cy3, Cy3B had a constant lifetime in various concentrations of glycerol solutions, showing that the lifetime of Cy3B is not influenced by adding glycerol to water (Supplementary Fig. S1b). In microdroplets, the fluorescence lifetime of Cy3B remained almost unaffected both at the periphery and the central region (τ_center_ = 2.2 ns and τ_periphery_ = 2.3 ns) compared to that in the bulk water (τ_bulk_ = 2.3 ns), as shown in Fig. 1c,e). These data confirm that the change in the lifetime of Cy3 observed in aqueous microdroplets was caused by the restriction of intramolecular rotation.

### Fluorescence lifetime lengthening extends over several microns from the droplet periphery

**Figure 2:**
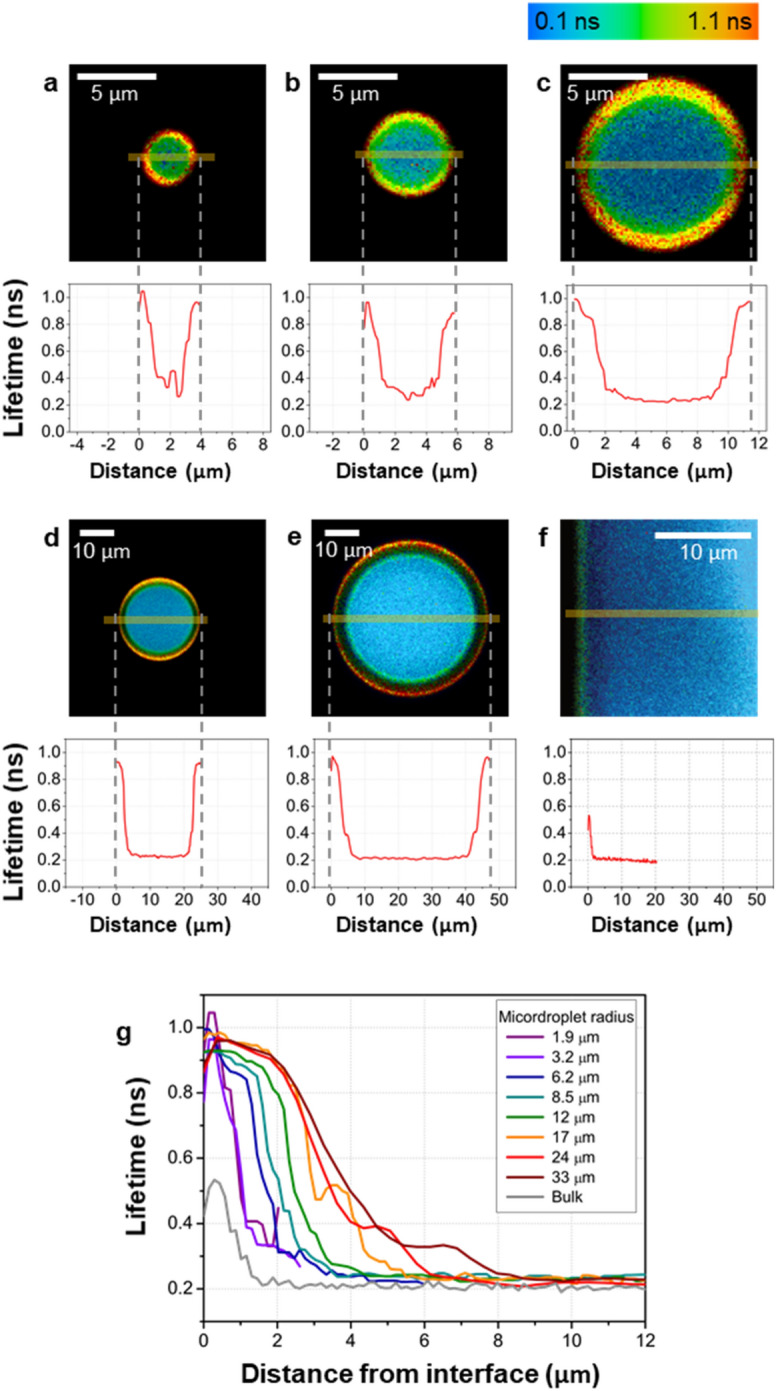
Effect of microdroplet size on Cy3 fluorescent lifetime at the periphery and center. (**a**–**f**) The FLIM images of aqueous microdroplets containing 1 μM Cy3 with various sizes (**a**–**e**) and of the bulk water–oil interface (**f**). The cross-sectional lifetimes of Cy3 are plotted below each FLIM image. (**g**) The cross-sectional profiles of the fluorescence lifetimes of Cy3 in various sizes of aqueous microdroplets and bulk water. The fluorescence lifetime is plotted against the distance from the oil–water interfaces of microdroplets with various radii and compared to that in bulk water.

Different sizes of aqueous microdroplets exert different effects on reactions occurring in microdroplets^3,8,12,13^. We thus explored if the spatial distribution of the fluorescence lifetime of Cy3 also depends on the microdroplet size. Figure 2a–f show the FLIM images for various microdroplet sizes (Fig. 2a–e) and of the bulk water–oil interface (Fig. 2f) along with their cross-sectional profiles of the lifetime. Figure 2g shows the comparison of the cross-sectional profiles of the fluorescence lifetime of Cy3 from the oil–water interface to the center of microdroplets for various microdroplet sizes and from the bulk-water–oil interface as a function of distance. These cross-sectional profiles clearly show that the fluorescence lifetime of Cy3 at the periphery of all microdroplets is longer than that at the bulk-water–oil interface. In addition, the peripheral region with lengthened fluorescence lifetime is larger in microdroplets than in bulk water. For example, for microdroplets with radii larger than approximately 17 µm, the peripheral region of lengthened fluorescence lifetime of Cy3 reaches over ~ 5 µm from the oil–water interface. In contrast, the region of lengthened fluorescence lifetime is less than 2 µm from the oil–water interface in the bulk solution. These results show that the peripheral region where the intramolecular rotation of Cy3 is restricted spans over several microns from the water–oil interface of aqueous microdroplets. We also observed that the peripheral region with the lengthened fluorescence lifetime increases as the microdroplet size increases (Supplementary Fig. S2). However, it is notable that the longest lifetimes of Cy3 remain essentially constant for different microdroplet sizes.

We argue that the peripheral region extending over 5 μm with the lengthened fluorescence lifetime is not originating from optical distortion, such as light diffraction or scattering effects. The 5-μm width of the peripheral field is far larger than the resolution limit of confocal microscopy^27^. In addition, the light diffraction and scattering do not affect the fluorescence lifetime that depends only on thermodynamic states of solvents and solvent-dye interactions. Consistent with this argument, the peripheral lifetime of Cy3 was independent of the size of microdroplets up to a radius of 33 μm (Fig. 2g). Therefore, we conclude that the influence of the water–oil interface of microdroplets affecting the intramolecular rotation of molecules extends over several microns.

We further confirmed this finding by carrying out a control experiment using FLIM imaging in microdroplets formed from reverse micelles. In reverse micelles, phospholipids form a monolayer interface between aqueous microdroplets and surrounding immersion oil. By staining the phospholipid layer with lipid-embedded dyes and imaging the location of dyes, we can examine whether any optical distortion appears in microdroplets and whether the lifetime lengthening observed over the range of several micron is influenced by any optical effect in microdroplets (Supplementary Fig. S3a). We prepared dual-stained reverse micelles with CellMask Green (Cell mask) and Alexa Fluor 488 (AF488) (Supplementary Fig. S3b). Cell mask dyes are lipid-embedded dyes; therefore, they are localized in a single layer between water and oil. AF488 dye molecules are water soluble and reside in the interior of reverse micelles. Because these dyes have distinctly different fluorescence lifetimes (2.5 ns for Cell mask dyes and 4.0 ns for AF488 dyes), distribution of these dyes in micelles can be readily determined with FLIM imaging of the micelles. Supplementary Figure S3c shows a FLIM image and a cross-sectional lifetime profile of a reverse micelle. Cell mask dyes that have 2.5 ns lifetime were exclusively located within the approximately 2-µm thickness of the micelle surface (green shaded region), which could be considered to be the diffraction-limited spatial resolution of the optical system used in this study. The observed approximately 5-µm thickness of the interfacial layer in which the lifetime is lengthened in microdroplets is distinguishably larger than this spatial resolution of the optics used. This result confirms that the spatial extent of the lifetime lengthening was not caused by optical dispersion or distortion brought about by the curved surface of a microdroplet.

We examined next whether this lengthening of fluorescence lifetimes is a unique property observable only in microdroplets but not in bulk water by comparing the fluorescence lifetime of Cy3 at the periphery of microdroplets to that of the bulk-water–oil interface. Although the bulk-water–oil interface also exhibited a lengthened fluorescence lifetime, the maximum value of increased lifetime at the bulk-water–oil interface was over two-fold lower than that of the microdroplet periphery (Fig. 2f). In addition, the thickness of the region where lifetime increased at the bulk-water–oil interface (approximately 1.5 μm) was relatively thinner than that of aqueous microdroplets (up to approximately 5 µm). These observations show that the effect on the restriction of the intramolecular rotation is enhanced and reached over a more extended range from the interface of microdroplets than from the bulk-water–oil interface.

We also investigated the change of the fluorescence lifetime of other fluorescent molecular rotors to examine whether similar behaviors were observed with different types of fluorescent dyes. Table 1 summarizes the results of comparing the fluorescence lifetime of Cy5 and AF555 between microdroplet and bulk water. Cy5 has a similar structure to Cy3 but has a conjugated five-carbon chain instead of a three-carbon between the two aromatic groups, which are able to rotate relative to each other (Supplementary Fig. S1a). Because of the longer chain length, the intramolecular rotation of Cy5 in bulk water is much slower than that of Cy3: 0.18 ns for Cy3 and 0.85 ns for Cy5 in the bulk water. The peripheral and central lifetimes of Cy5 in oil-confined water microdroplets were measured to be 1.2 and 0.86 ns, respectively. Although the effect of microdroplets for Cy5 on its rotation was not as pronounced as Cy3 (τ_periphery_ = 1.2 ns for Cy5 and τ_periphery_ = 0.86 ns for Cy3) owing to the slower intrinsic rotation rate of Cy5 dye molecules, Cy5 exhibited increased fluorescence lifetime at the periphery of water microdroplets compared to that in the bulk water (τ_bulk_ = 0.85 ns). ATTO 647 N dye, which has a similar fluorescence spectrum to Cy5 but with a rigid backbone (Supplementary Fig. S1a), showed nearly constant fluorescence lifetimes both in the periphery and central regions (τ_center_ = 3.5 ns; τ_periphery_ = 3.2 ns), which is similar to that (τ_bulk_ = 3.5 ns) in bulk water. These observations again confirm that the lengthened fluorescence lifetime is caused by the restriction of intramolecular rotation.

**Table 1:** The fluorescence lifetime of the molecular internal rotors.

Classification	Fluorescent dyes	Fluorescence lifetime (ns) ± SD*		
		Microdroplets		Bulk
		Periphery	Center	
Internal rotor molecules	Cy3	0.86 ± 0.052	0.17 ± 0.012	0.18 ± 0.0029
	Cy5	1.2 ± 0.13	0.86 ± 0.11	0.85 ± 0.031
	AF555	0.47 ± 0.051	0.29 ± 0.0091	0.32 ± 0.0031
Rigid molecules	Cy3B	2.2 ± 0.034	2.3 ± 0.051	2.3 ± 0.0089
	ATTO647N	3.2 ± 0.29	3.5 ± 0.12	3.5 ± 0.055

*SD standard deviation from more than ten and five independent measurements for microdroplets and for bulk, respectively.

In addition, we tested the AF555 rotor, an analog of Cy3, which has 4 sulfonate groups in rotating wing residues instead of 2 sulfonate groups in Cy3 (Supplementary Fig. S1a). The lifetime of AF555 at the microdroplet periphery increased to 0.47 ns compared to 0.32 ns in the bulk water and 0.29 ns at the microdroplet center. These measurements support that the behavior of the restricted intramolecular rotation at the periphery of aqueous microdroplets does not arise from the property of a particular dye but is a general characteristic of intramolecular rotor dyes dissolved in water microdroplets surrounded by oil.

We examined any possible effect of Cy3 concentration on the lifetime. Supplementary Figure S3a shows the fluorescence lifetimes of Cy3 in bulk solutions containing different concentrations of Cy3. There was almost no change in the lifetime of Cy3 with concentrations over the range from 430 nM to 1 mM, confirming that the lifetime of Cy3 is mostly independent of the dye concentration. The Cy3 molecules tend to be enriched at the interface of water microdroplets under our experimental conditions (Figs. 1b, 2a–f, and Supplementary Fig. S4b). Supplementary Figure S4b, c present the spatial profile of the Cy3 intensity and lifetime, which does not directly match (Supplementary Fig. S4d). Both results confirmed that the lifetime lengthening of Cy3 observed in microdroplets is not caused by the dye concentration at the interface. Moreover, we measured the efficiency of Förster resonance energy transfer (FRET) between Cy3 and Cy5 to test the possibility that an intermolecular interaction may affect the lifetime of Cy3. At both the periphery and the center of microdroplets, the FRET efficiency value remained the same as that in bulk water (Supplementary Fig. S5). These data also support that intermolecular distances between dye molecules are not close enough to cause an intermolecular interaction and that the lifetime lengthening observed at the periphery of microdroplets is not caused by intermolecular interaction between Cy3 dye molecules.

### Lifetime lengthening is observed only in water microdroplets

**Figure 3:**
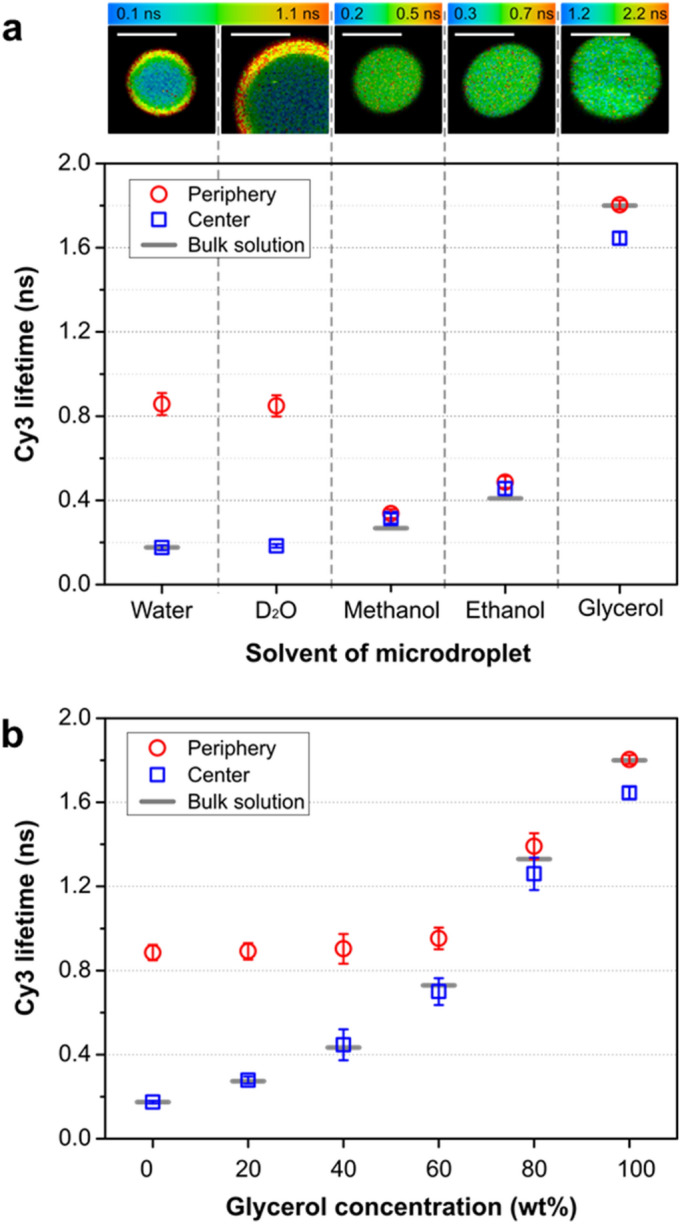
(**a**) Lifetimes of Cy3 in microdroplets made of water, D_2_O, methanol, ethanol, and glycerol. Representative images of fluorescence lifetime containing each solvent are shown. Scale bar, 5 µm. (**b**) Lifetimes of Cy3 at the peripheral (red circle) and central regions (blue square) of aqueous microdroplets containing various concentrations of glycerol. The lifetimes in bulk solution measured in each condition are marked with gray lines. Error bars represent one standard deviation from more than ten independent measurements.

We then asked if the lengthening of fluorescence lifetime is a unique property of microdroplets of water molecules or commonly observed in microdroplets of other organic solvent molecules. For this test, we compared the fluorescence lifetime in microdroplets made of water with those of methanol, ethanol, and glycerol. We generated these microdroplets that have a different solvent composition by sonicating the mixture of each solvent containing 1-µM Cy3 dyes and immersion oil (1:10, v/v). We found that the lifetimes both in the microdroplet periphery and the microdroplet center remained almost the same as that of the bulk water for all of the tested organic solvents (Fig. 3a). This result shows that the lengthening of fluorescence lifetime is observed only in water microdroplets. In addition, the intramolecular rotational behavior of Cy3 in microdroplets made of D_2_O was very similar to that in microdroplets of H_2_O, showing that intramolecular rotation is not influenced by the heavy isotope, deuterium.

We next tested if different types of oils surrounding water microdroplets affect the observed fluorescence lifetime changes of Cy3 dyes in microdroplets. For this study, we carried out FLIM imaging of aqueous microdroplets surrounded by silicone oil (Supplementary Fig. S6). We observed a similar behavior of lifetime lengthening of Cy3 dyes at the periphery of microdroplets (τ_periphery_ = 0.64 ns) compared to that of the center of microdroplets (τ_center_ = 0.17 ns) and the bulk water (τ_bulk_ = 0.18 ns). However, it is noted that the peripheral lifetime in water-in-silicone oil microdroplets (0.64 ns) was shorter than the one in water-in-immersion oil microdroplets (0.86 ns). This result might be caused by a change in the size of the electric field at the interface, the distribution of water ions, and the change in the strength of hydrogen bonding between water molecules or water-dye molecules, which are discussed below. However, the thickness of the interfacial layer in which the lifetime lengthening occurs did not significantly vary between the two different types of oils. This invariant width between two oils that have different refractive indices (1.479 for silicone oil and 1.515 for immersion oil) also confirms that little optical distortion occurred in the imaging measurements.

### Possible origin of the restricted intramolecular rotation in aqueous microdroplets

The increase of fluorescence lifetime of internal rotor molecules is often caused by the increase of solvent viscosity^23^. We examined whether the increase of fluorescence lifetime was caused by the increased local viscosity at the periphery of microdroplets. Glycerol lengthens fluorescence lifetime of intramolecular rotors by increasing the viscosity of solution^25^. We added different concentrations of glycerol in aqueous microdroplets and measured fluorescence lifetime of Cy3. The lifetime in the central region of microdroplets increased as the concentration of glycerol increased with a trend similar to that in the bulk water solution (Fig. 3b). However, the fluorescence lifetime of Cy3 at the periphery did not change significantly as the glycerol concentration was increased up to 60%. Thus, the restricted intramolecular rotation observed at the periphery of microdroplets was not additive to the increased viscosity of glycerol solution up to 60% in this experiment. The result suggests that the increase of solution viscosity derived from glycerol may not be enough to explain the lifetime lengthening observed in microdroplets, and there may exist other mechanisms.

Multiple possible explanations can be offered for the observed lengthened fluorescence lifetime at the microdroplet periphery. The fluorescence lifetime of molecules undergoing intramolecular rotation is influenced by several factors, including solution viscosity, pH, solvent composition, electric field, redox status, and temperature^25^. First, local pH change formed in microdroplets can be a cause of the lifetime increase. It has been reported that there is a gradient of pH across microdroplets^28,29^, which can be caused by the preferred adsorption of hydroxide ions^30,31^, leading to an increase of pH, at the interface of the water-hydropic medium. We examined the effect of pH on the lifetime of Cy3 in bulk water solution. Supplementary Figure S7 shows that the lifetimes of Cy3 in bulk water solution do not change under different pH values in the range between pH 2.0 and pH 12.3. This result is consistent with a previous observation that the lifetime of cyanine dye is independent of the solution pH in the range of pH 2.5–11^32^. Thus, the lengthening of fluorescence lifetime at the periphery of a microdroplet is unlikely caused by the local pH change.

Second, enhanced hydrogen bonding between water molecules at the interface of water and oil may be responsible for the lifetime change in microdroplets. It was reported that a stronger hydrogen bond between water molecules forms at the interface of nanodroplets compared to the planar interface between water and hydrophobic liquid^14^. This enhanced hydrogen bonding between water molecules and water to dye molecules can restrict the rotational movement of dye molecules and induces the lengthening of fluorescence lifetime. Third, the electric field formed at the water-hydrophobic interface^18–22^ may be responsible for the lifetime change by aligning water molecules and dye molecules, and restricting intramolecular rotations. In previous studies^33–36^, the interfacial effects were observed only within several atomic layers to several nanometers distance from the heterogeneous interface. In contrast, the interfacial effect on restricting intramolecular rotation that we found in microdroplets extends over several microns. At first this result may seem surprising, but calculations indicate that the electric field arising from the electric double layer at the water–oil interface can extend deeply into the microdroplet^22^, which supports this possible mechanism. Besides the above possibilities, surface tension can be considered as another contributing factor to the restricted intramolecular rotation in aqueous microdroplets. Surface tension arises from the cohesive force of solvent molecules. The stronger hydrogen bond and thus a stronger surface tension between water molecules at the interface of microdroplets than that at the planar interface of bulk water may contribute to the lengthened fluorescence lifetime of Cy3 at the periphery of microdroplets. This speculation is supported by the observation that the peripheral lifetime of microdroplets made of water and glycerol with relatively higher surface tension has a longer fluorescence lifetime compared to ethanol and methanol with relatively lower surface tension (Fig. 3b).

Clearly, more work is needed to assess the relative importance of these different contributions to fluorescence lifetime lengthening, but the present study firmly establishes that the fluorescence lifetime of internal-rotor dyes is lengthened over the range of a few microns from the water–oil interface. Another notable observation was that the depth of the lengthened lifetime is expanded with the size of droplet (Fig. 2g, Supplementary Fig. S2). Presently, we do not have a clear understanding of why the thickness of the interfacial layer with lengthened lifetime increases as the microdroplet size increases and what is the nature of this long-range layer. A clue may come from the cross-sectional profiles of the lifetime. The profiles in small microdroplets with radii less than 3.2 μm are simple. There is a lifetime peak at the interface, which then sharply decreases toward the center of the microdroplet. The profiles become different from microdroplets with over 6.2 μm in radii, where a lifetime peak becomes broad and then there is a slow decrease of the lifetime profiles. It is also noted that the slope of the decrease becomes less steep as the microdroplet size increases. This observation may be interpreted in the following way. The water–oil interface of the microdroplets has a potential to influence the solvent characteristics of water over approximately 5 μm. This potential can be manifested as broad peaks in large microdroplets with less steep decrease in a larger microdroplets but results in a sharp decrease toward the center in small microdroplets. We suspect that the manifestation of the interface potential into a broad peak in large microdroplets may have a limit, as the broadening of the lengthened lifetime profiles started to slow down for microdroplets with 17 μm in radius or larger (Supplementary Fig. S2).

## Conclusion

We report the lengthened lifetime of fluorescent molecular rotors, including Cy3, Cy5, and AF555, at the periphery of aqueous microdroplets enclosed in oils. Compared to the planar bulk water–oil interface, the lifetime lengthening effect was much enhanced in microdroplets, which was observed to extend over several microns from the water–oil interface of the microdroplets. The lengthening of fluorescence lifetime occurred only in aqueous microdroplets, not in microdroplets made of organic solvents. These results show that the water microdroplet environment provides a unique environment for the restriction of intramolecular rotation. These observations offer supporting evidence for the minimized entropy in chemical reactions in aqueous microdroplets, which can enable the occurrence of thermodynamically unfavorable reactions. In general, the observations reported here support the idea that aqueous microdroplets provide a unique chemical and biochemical environment.

## Materials and methods

### Preparation of water-in-oil microdroplet

The mono NHS ester fluorescent dyes were purchased from Lumiprobe (Cy3 and Cy5), ThermoFisher Scientific (Alexa Fluor 555), GE Healthcare (Cy3B), and ATTO-TEC (ATTO 647 N). The stock solutions were prepared at 1 mM concentration and diluted in distilled water (Milli-Q Integral 15 Systems, Millipore, France) to the final concentration of 1 µM for FLIM measurements.

For the imaging of microdroplet, aqueous microdroplets were prepared by sonicating the mixture of 5 µL distilled water containing each fluorescent dye (1 µM) and 50 µL the immersion oil (type A, Nikon, Japan) in a bath sonicator (Elmasonic S 10; Elma Schmidbauer GmbH, Germany) with ‘sweep’ function for 1 min. The prepared oil-solution emulsion was placed onto a coverslip and imaged with a confocal microscope (Leica TCS SP8 X, Germany) equipped with a FLIM measuring system (PicoQuant, Germany).

### FLIM imaging of microdroplets and image analysis

The solution containing microdroplets in oil was placed onto a coverslip and imaged with a confocal microscope (HC PL APO CS2 63x/1.40 OIL, Leica SP8). The fluorophores were excited by the laser beams (20 MHz 540 nm for Cy3, Cy3B, and AF555; and 20 MHz 640 nm for Cy5 and ATTO 647 N) and detected by HyD SMD (550–750 nm and 650–750 nm, respectively). For FLIM measurements, the scan speed was 10 Hz and the pixel size was 0.145 nm or 0.362 nm depending on the microdroplet size.

For the FLIM measurement of the bulk solution, 20 μL of dye solution was placed on a coverslip, which was surrounded by the immersion oil. The FLIM imaging was carried out near the interface between the bulk water and the bulk oil.

To analyze the lifetime of the peripheral and the central region of microdroplets, the region of interest (ROI) was set in SymPhoTime software (Picoquant). Enough counts of photons in each ROI were acquired for a statistically meaningful fitting of fluorescence decay patterns to exponential curves. The decay curves were fitted by either a single- or bi-exponential function, depending on the shape of the decay curves. For the analyses of FLIM of Cy3 in an aqueous microdroplet, fluorescence decay curves in each pixel were fitted using a bi-exponential tail fit using SymPhoTime. Lifetime images were processed with ImageJ (NIH, Bethesda, MD, USA). We measured the central fluorescence lifetime at the concentric region of microdroplets with a radius of 1–5 μm depending on the microdroplet size, where lifetimes were kept at relatively constant values. The peripheral lifetime was measured from outermost regions of up to the inflection point of the lifetime curves.

In order to examine the effect of solvent composition on the fluorescence lifetime of Cy3 in both bulk solution and microdroplets, the Cy3 stock solution was diluted in methanol, ethanol, deuterium oxide (Merck, Germany), or glycerol (LPS Solution, Korea). FLIM imaging of these microdroplets was conducted in the same way as for aqueous microdroplets.

### FLIM imaging of dual-stained reverse micelles

The reverse micelles were prepared by emulsifying the mixture of lipid-containing oil and aqueous solution. After making dry films of POPC (1-palmitoyl-2-oleoylphosphatidylcholine, Avanti Polar Lipids, AL, USA) and Cellmask on the bottom of a glass vial under nitrogen gas perfusion, the immersion oil was added to the dry film to make the final concentration of 1 mM POPC and 1 × Cellmask. To complete the solvation, the mixture was sonicated for 1 h at 50 °C in the bath sonicator. Then, 1 μM AF488 in distilled water was added to the oil-mixture with 1:10 ratio. The micelle formation through bath sonication was done in the same way as the water-in-oil droplet formation.

### The FRET efficiency between Cy3 and Cy5 in microdroplets and the bulk water

Cy3 and Cy5 at the concentration of 0.5 μM were used as a donor and an acceptor, respectively. Fluorescence signals from the donor (540–640 nm) and the acceptor (650–750 nm) were measured upon 530 nm excitation using confocal microscope (Leica TCS SP8 X, Germany). The FRET efficiency is then calculated as the following ratio: *F*_*a*_*/*(*F*_*d*_ + *F*_*a*_), where *F*_*a*_ is the acceptor emission and *F*_*d*_ is the donor emission in each defined ROI region.

## Supplementary information

Supplementary information.

## References

[1] Lee JK, Kim S, Nam HG, Zare RN (2015). Microdroplet fusion mass spectrometry for fast reaction kinetics. Proc. Natl. Acad. Sci. USA.

[2] Lee JK, Nam HG, Zare RN (2017). Microdroplet fusion mass spectrometry: accelerated kinetics of acid-induced chlorophyll demetallation. Q. Rev. Biophys..

[3] Fallah-Araghi A (2014). Enhanced chemical synthesis at soft interfaces: a universal reaction–adsorption mechanism in microcompartments. Phys. Rev. Lett..

[4] Li Y, Yan X, Cooks RG (2016). The role of the interface in thin film and droplet accelerated reactions studied by competitive substituent effects. Angew. Chem. Int. Ed. Engl..

[5] Bain RM, Pulliam CJ, Cooks RG (2015). Accelerated Hantzsch electrospray synthesis with temporal control of reaction intermediates. Chem. Sci..

[6] Lee JK, Banerjee S, Nam HG, Zare RN (2015). Acceleration of reaction in charged microdroplets. Q. Rev. Biophys..

[7] Zhang WW, Cheng HY, Liu JH (2018). Accelerated two-phase oxidation in microdroplets assisted by light and heat without the use of phase-transfer catalysts. ACS Sustain. Chem. Eng..

[8] Lai YH, Sathyamoorthi S, Bain RM, Zare RN (2018). Microdroplets accelerate ring opening of epoxides. J. Am. Soc. Mass. Spectrom..

[9] Nam I, Lee JK, Nam HG, Zare RN (2017). Abiotic production of sugar phosphates and uridine ribonucleoside in aqueous microdroplets. Proc. Natl. Acad. Sci. USA.

[10] Nam I, Nam HG, Zare RN (2018). Abiotic synthesis of purine and pyrimidine ribonucleosides in aqueous microdroplets. Proc. Natl. Acad. Sci. USA.

[11] Lee JK, Samanta D, Nam HG, Zare RN (2019). Micrometer-sized water droplets induce spontaneous reduction. J. Am. Chem. Soc..

[12] Lee JK (2019). Spontaneous generation of hydrogen peroxide from aqueous microdroplets. Proc. Natl. Acad. Sci. USA.

[13] Lee JK, Samanta D, Nam HG, Zare RN (2018). Spontaneous formation of gold nanostructures in aqueous microdroplets. Nat. Commun..

[14] Smolentsev N, Smit WJ, Bakker HJ, Roke S (2017). The interfacial structure of water droplets in a hydrophobic liquid. Nat. Commun..

[15] Zhou Z, Yan X, Lai YH, Zare RN (2018). Fluorescence polarization anisotropy in microdroplets. J. Phys. Chem. Lett..

[16] Hosny NA (2013). Fluorescent lifetime imaging of atmospheric aerosols: a direct probe of aerosol viscosity. Faraday Discuss..

[17] Hosny NA (2013). Mapping microbubble viscosity using fluorescence lifetime imaging of molecular rotors. Proc. Natl. Acad. Sci. USA.

[18] Kathmann SM, Kuo IF, Mundy CJ (2008). Electronic effects on the surface potential at the vapor–liquid interface of water. J. Am. Chem. Soc..

[19] Cendagorta JR, Ichiye T (2015). The surface potential of the water-vapor interface from classical simulations. J. Phys. Chem. B.

[20] Leung K (2010). Surface potential at the air–water interface computed using density functional theory. J. Phys. Chem. Lett..

[21] Kwan V, Consta S (2020). Bridging electrostatic properties between nanoscopic and microscopic highly charged droplets. Chem. Phys. Lett..

[22] Chamberlayne CF, Zare RN (2020). Simple model for the electric field and spatial distribution of ions in a microdroplet. J. Chem. Phys..

[23] Athanasiadis A (2016). Dynamic viscosity mapping of the oxidation of squalene aerosol particles. Phys. Chem. Chem. Phys..

[24] Jia K (2007). Characterization of photoinduced isomerization and intersystem crossing of the cyanine dye Cy3. J. Phys. Chem. A.

[25] Kuimova MK, Yahioglu G, Levitt JA, Suhling K (2008). Molecular rotor measures viscosity of live cells via fluorescence lifetime imaging. J. Am. Chem. Soc..

[26] Cooper M (2004). Cy3B: improving the performance of cyanine dyes. J. Fluoresc..

[27] Suhling K (2015). Fluorescence lifetime imaging (FLIM): Basic concepts and some recent developments. Med. Photonics.

[28] Wei H (2018). Aerosol microdroplets exhibit a stable pH gradient. Proc. Natl. Acad. Sci. USA.

[29] Beattie JK, Djerdjev AM, Warr GG (2009). The surface of neat water is basic. Faraday Discuss.

[30] Zangi R, Engberts JB (2005). Physisorption of hydroxide ions from aqueous solution to a hydrophobic surface. J. Am. Chem. Soc..

[31] Bai C, Herzfeld J (2016). Surface propensities of the self-ions of water. ACS Cent. Sci..

[32] Berezin MY (2011). Near-infrared fluorescence lifetime pH-sensitive probes. Biophys. J..

[33] Chowdhary J, Ladanyi BM (2006). Water–hydrocarbon interfaces: effect of hydrocarbon branching on interfacial structure. J. Phys. Chem. B.

[34] Bresme F, Chacon E, Tarazona P, Tay K (2008). Intrinsic structure of hydrophobic surfaces: the oil–water interface. Phys. Rev. Lett..

[35] Bjorneholm O (2016). Water at interfaces. Chem. Rev..

[36] Mitrinovic DM, Tikhonov AM, Li M, Huang Z, Schlossman ML (2000). Noncapillary-wave structure at the water–alkane interface. Phys. Rev. Lett..

