# Supplementary material for: Restricted intramolecular rotation of fluorescent molecular rotors at the periphery of aqueous microdroplets in oil
Source: Sci Rep. 2020 Oct 8;10:16859. doi: 10.1038/s41598-020-73980-7 (PMC7545199; doi:10.1038/s41598-020-73980-7)
Supplement: Supplementary file 1 — Supplementary information. [file 41598_2020_73980_MOESM1_ESM.docx]

Supplementary Information for

Restricted Intramolecular Rotation of Fluorescent Molecular Rotors at the Periphery of Aqueous Microdroplets in Oil

Jooyoun Kang^1,2†^, SangMoon Lhee^1†^, Jae Kyoo Lee^2^, Richard N. Zare^2^*, and Hong Gil Nam^1,3^*

^1^ Center for Plant Aging Research, Institute for Basic Science, Daegu 42988, Republic of Korea.

^2^ Department of Chemistry, Stanford University, Stanford, CA 94305 USA.

^3^ Department of New Biology, DGIST, Daegu 42988, Republic of Korea.

^†^ These authors equally contributed to this work.

* Corresponding authors: nam@dgist.ac.kr, [zare@stanford.edu](mailto:zare@stanford.edu)


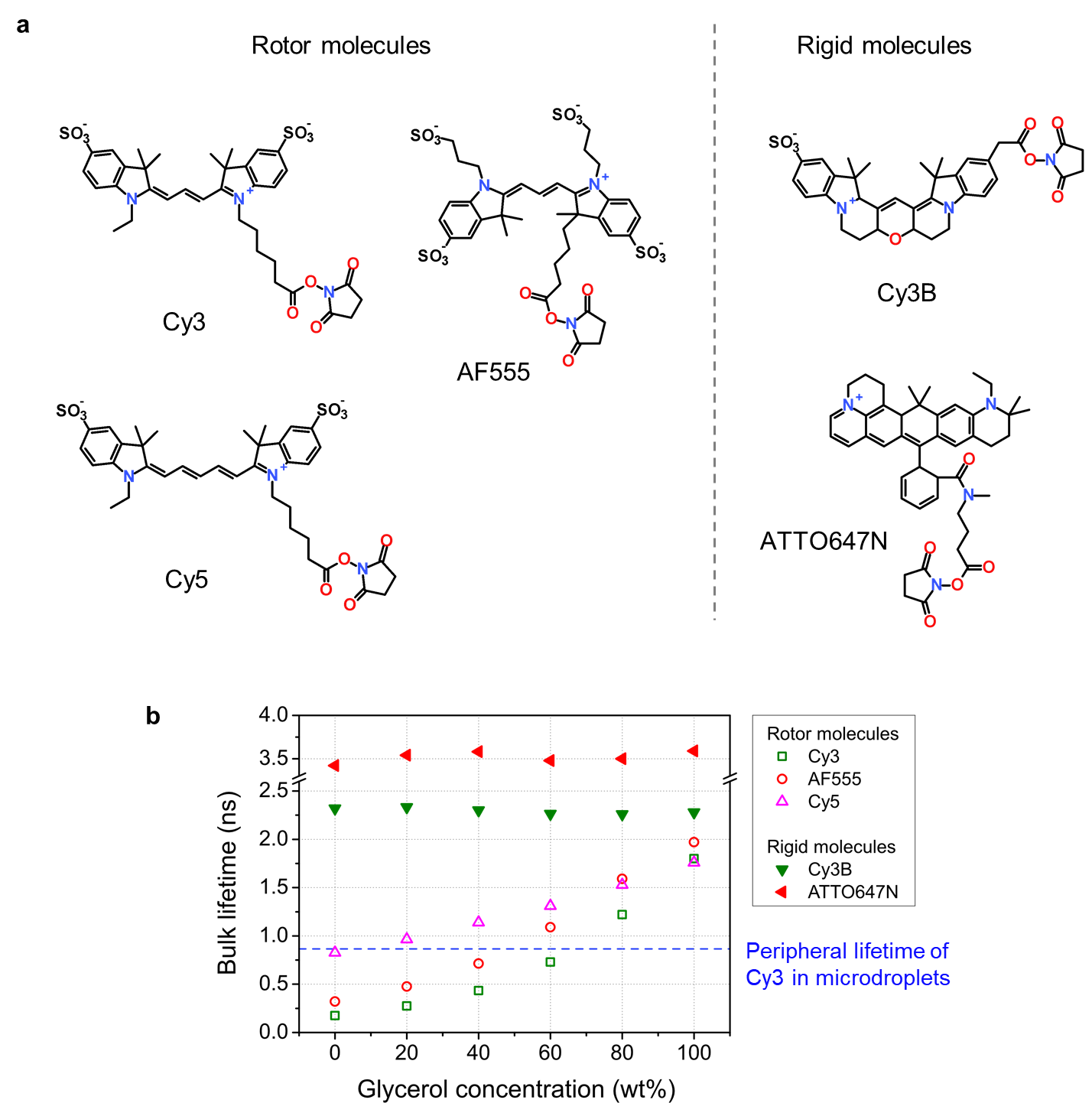


**Figure S1.** Chemical structures of fluorescence dyes used for experiments and the change of fluorescence lifetimes of these dyes in different concentrations of glycerol solution. (**a**) Cy3, Cy5, and Alexa Fluor 555 (AF555) have conjugated chains between aromatic groups that are able to rotate relative to each other, whereas Cy3B and ATTO647N have rigid backbones that do not allow rotation of the aromatic groups. All these dyes contain the NHS functional group. (**b**) The lifetimes of the rotor molecules, Cy3, AF555, and Cy5, as well as Cy3B and ATTO647N, the rigid and Cy5, were measured in the bulk water containing different concentrations of glycerol. The lifetimes of rotor molecules were lengthened as the glycerol concentration increases, whereas those of rigid molecules presented constant values independent of different glycerol concentrations. The concentration of each fluorophore was 1 μM. The dashed blue line indicates the lifetime value of Cy3 at the microdroplet periphery.


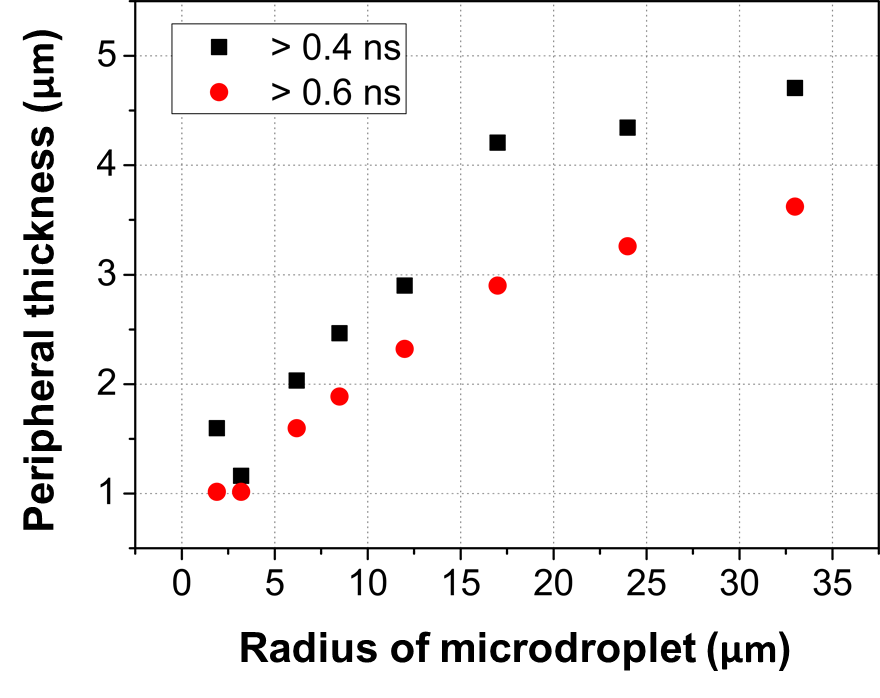


**Figure S2**. The breadth of the lengthened lifetime profiles. The peripheral thickness is the distance from the interface to the cross-sectioning distance with the lifetime of 0.4 or 0.6 ns. The peripheral thickness increases almost linearly with the microdroplet radius up to 17 µm, and then the increase rate becomes less afterward.


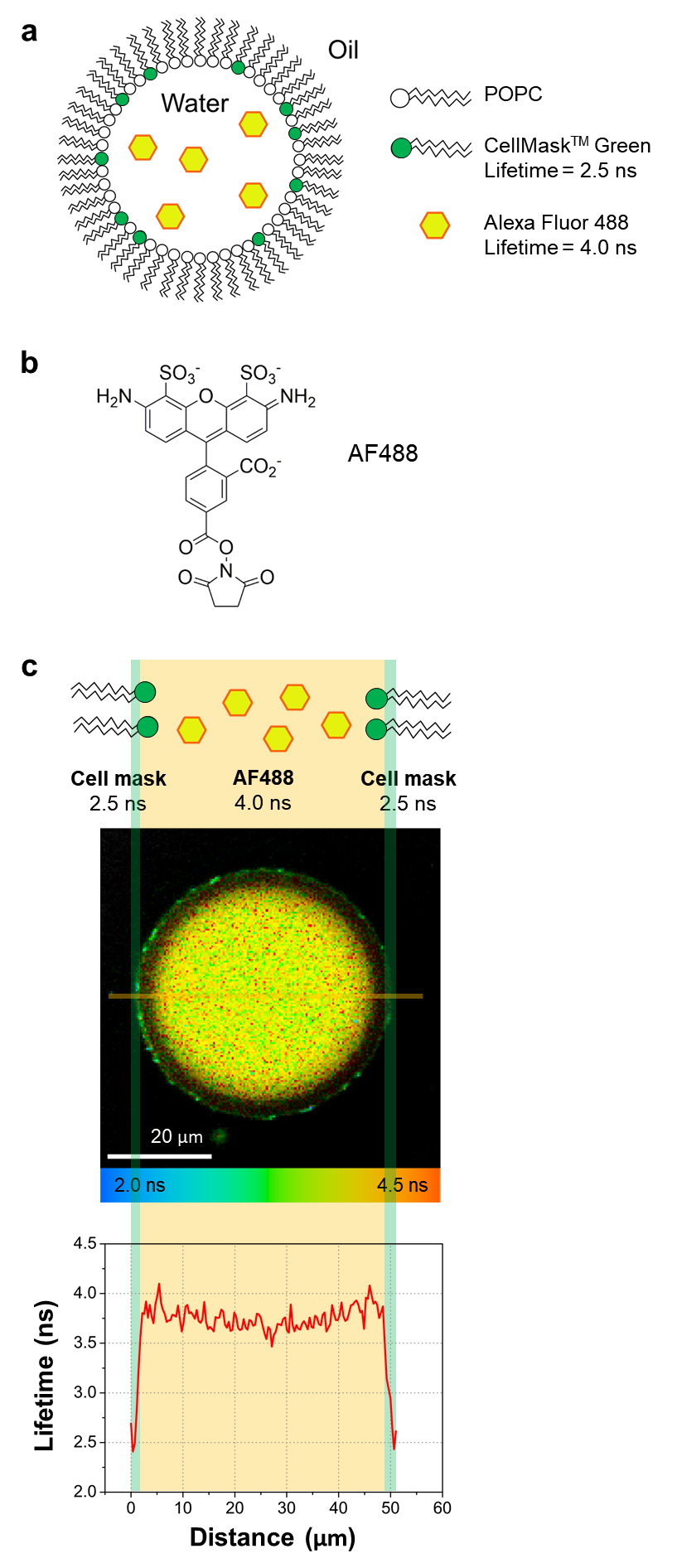


**Figure S3.** The fluorescence lifetime image of a reverse micelle stained with two different dyes that have distinguishable fluorescence lifetimes. (**a**) The experimental scheme of the reverse micelle stained with CellMask Green (Cell mask) and Alexa Fluor 488 (AF488). The reverse micelle was made of 1-palmitoyl-2-oleoylphosphatidylcholine (POPC) lipid that forms the interface between water and oil. The Cell mask dyes were embedded in the POPC single layer of the reverse micelle. Water-soluble AF488 dyes are located in the interior of reverse micelles. (**b**) Chemical structure of the water-soluble dye, AF488. Due to the rigid structure, AF488 exhibited a constant lifetime in microdroplets. (**c**) The image and the cross-sectional profile of the fluorescence lifetime of a reverse micelle dual-stained with Cell mask dyes and AF488 dyes. Cell mask dyes that have 2.5-ns lifetime were located within approximately 2 µm near the POPC layer of the micelle (green shaded region). AF488 dyes were in the interior of the reverse micelle (yellow shaded region).


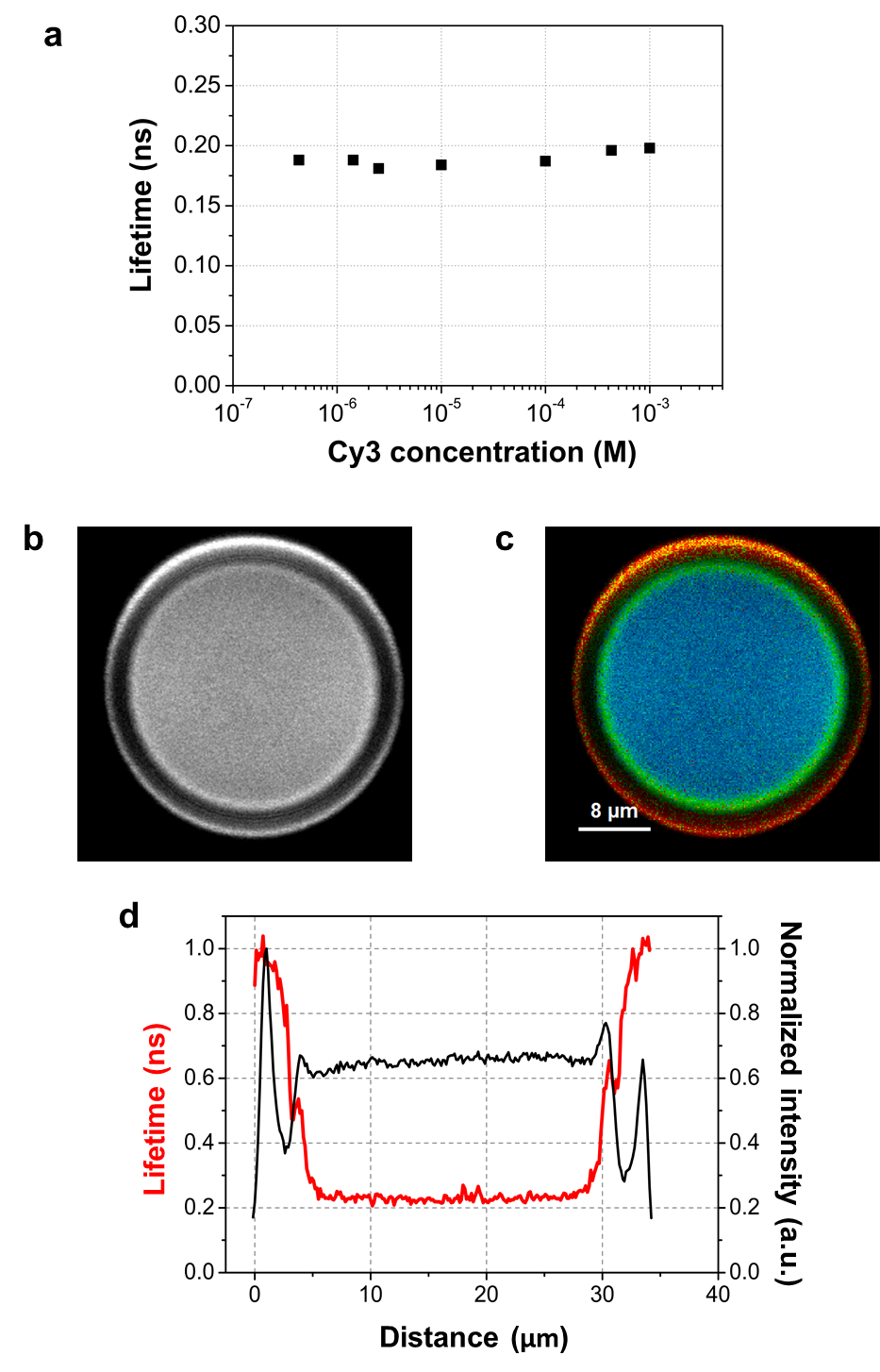


**Figure S4.** The independence of fluorescence lifetime to the concentration of the fluorophore. (**a**) The fluorescence lifetimes of Cy3 in the bulk water containing different concentrations of Cy3, ranging from 430 nM to 1 mM. The lifetimes of Cy3 remained almost constant at approximately 0.18 ns. (**b** and **c**) Fluorescence intensity image (**b**) and FLIM image (**c**) of Cy3 in an aqueous microdroplet encapsulated in immersion oil. (**d**) The cross-sectional profile of fluorescence lifetime (red) and intensity (black) in an aqueous microdroplet. Note that the two profiles are not directly correlated.


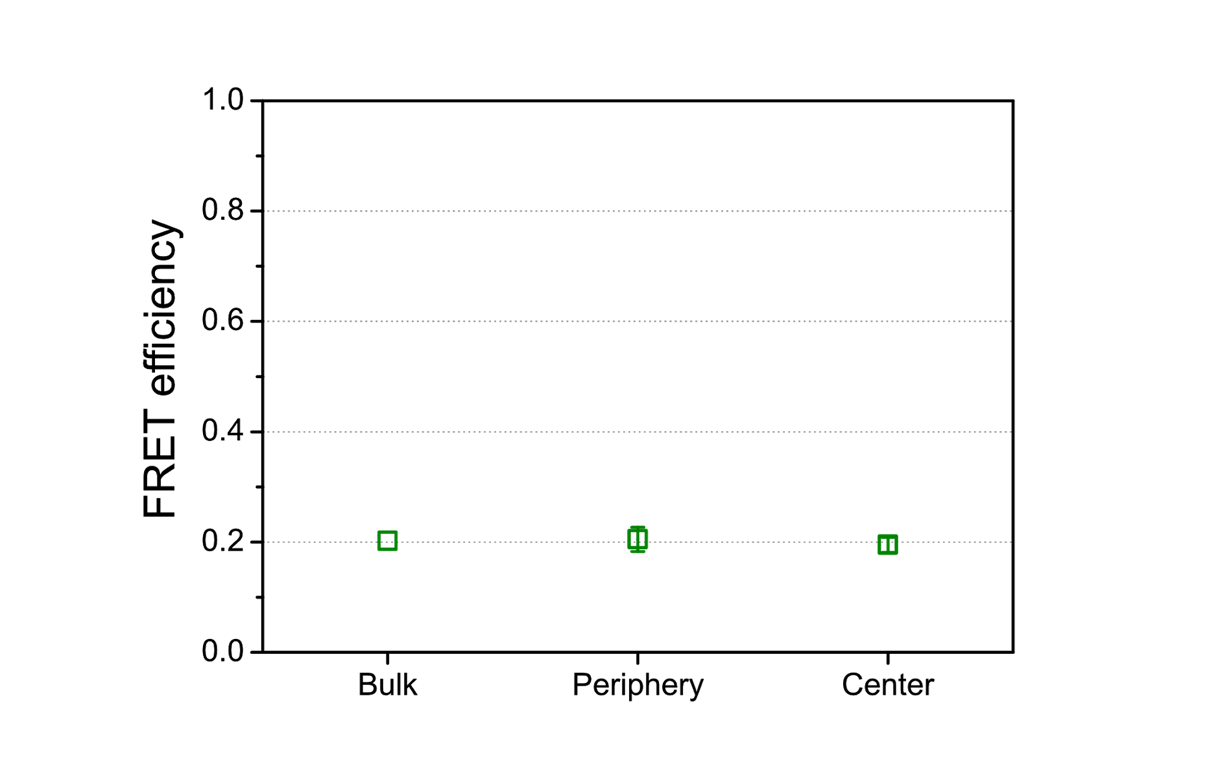


**Figure S5.** Förster resonance energy transfer (FRET) efficiency between Cy3 and Cy5 in bulk water and at the periphery and the center of microdroplets. In bulk solution, FRET efficiency is expected to be 0 owing to the low concentrations of dyes but was measured to be 0.2 in our experiment. This base-level FRET efficiency was caused by the direct excitation of acceptors and the leakage of the donor emission into the acceptor channel. The FRET efficiencies measured both at the periphery and the center of microdroplets were the same as in bulk.

**
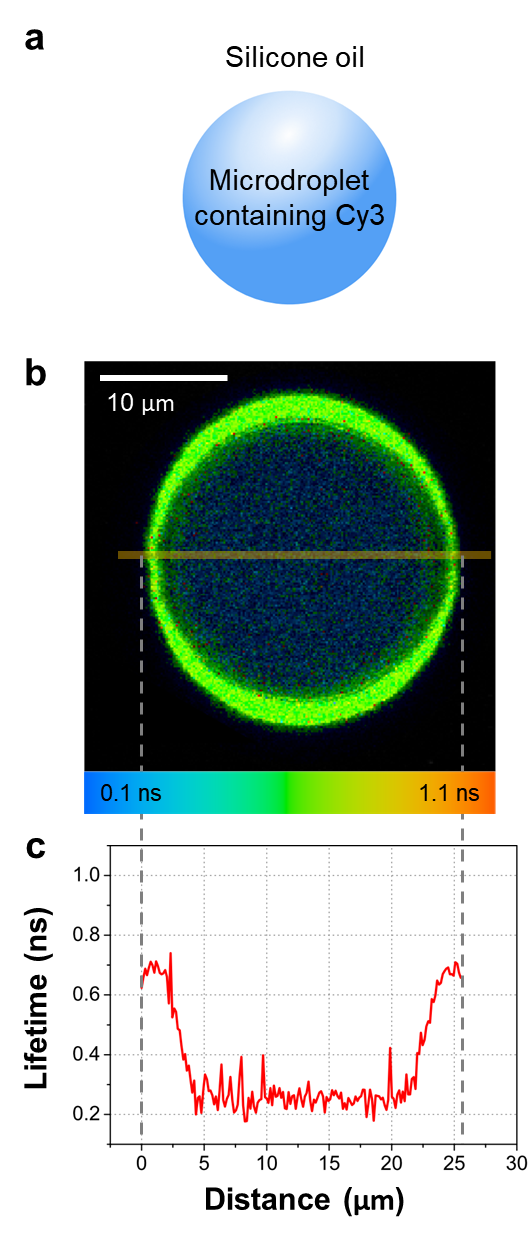
**

**Figure S6.** FLIM image of Cy3 dyes in aqueous microdroplets surrounded by silicone oil. (**a**) Experimental scheme of a microdroplet encapsulated by silicone oil. The fluorescence lifetime image (**b**) and the distributional profile (**c**) of Cy3 dyes in water-in-silicone oil show similar behavior of the fluorescence lifetime lengthening at the periphery of microdroplets observed in water-in-immersion oil.


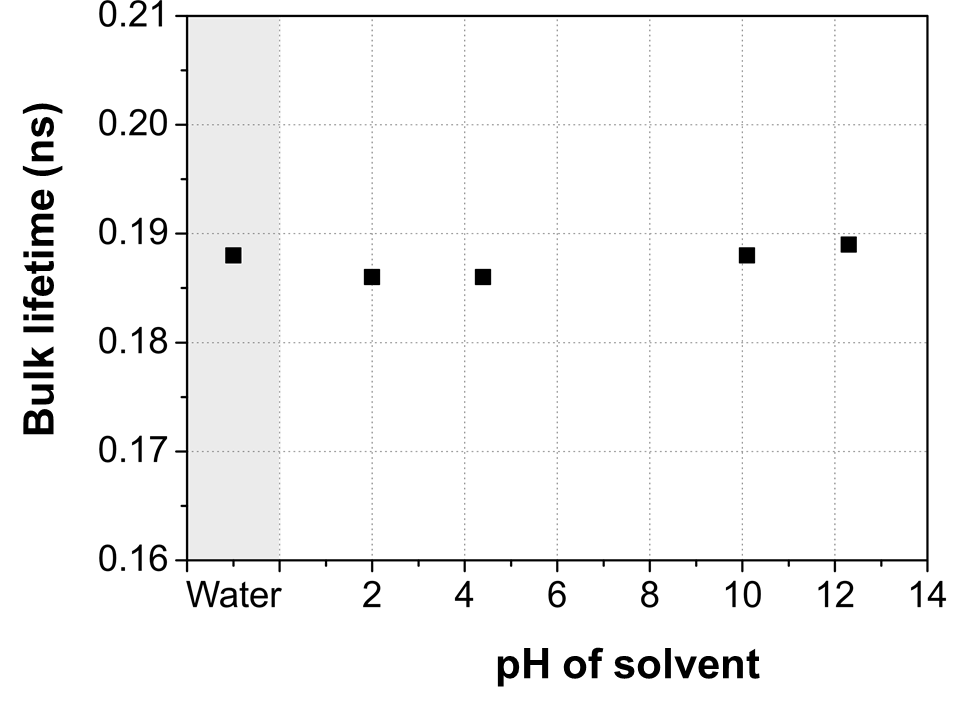


**Figure S7**. The fluorescence lifetime of Cy3 in bulk water solution with varying pH values. The fluorescence lifetime of Cy3 molecules was examined by dissolving dyes at the final concentration of 1 μM with pH adjusted to 2.0, 4.4, 10.1, and 12.3. The acidic and basic solvents were made using hydrogen chloride and sodium hydroxide solutions, respectively.
